# Hepatic HDAC3 Regulates Systemic Iron Homeostasis and Ferroptosis via the Hippo Signaling Pathway

**DOI:** 10.34133/research.0281

**Published:** 2023-11-30

**Authors:** Hongen Meng, Yingying Yu, Enjun Xie, Qian Wu, Xiangju Yin, Bin Zhao, Junxia Min, Fudi Wang

**Affiliations:** ^1^ The Second Affiliated Hospital, The First Affiliated Hospital, Institute of Translational Medicine, School of Public Health, Zhejiang University School of Medicine, Hangzhou, China.; ^2^ Institute of Emergency Management, Henan Polytechnic University, Jiaozuo, China.; ^3^ MOE Key Laboratory of Biosystems Homeostasis and Protection, Zhejiang Provincial Key Laboratory for Cancer Molecular Cell Biology, and Innovation Center for Cell Signaling Network, Life Sciences Institute, Zhejiang University, Hangzhou 310058, China.

## Abstract

Histone deacetylases (HDACs) are epigenetic regulators that play an important role in determining cell fate and maintaining cellular homeostasis. However, whether and how HDACs regulate iron metabolism and ferroptosis (an iron-dependent form of cell death) remain unclear. Here, the putative role of hepatic HDACs in regulating iron metabolism and ferroptosis was investigated using genetic mouse models. Mice lacking *Hdac3* expression in the liver (*Hdac3*-LKO mice) have significantly reduced hepatic *Hamp* mRNA (encoding the peptide hormone hepcidin) and altered iron homeostasis. Transcription profiling of *Hdac3*-LKO mice suggests that the Hippo signaling pathway may be downstream of Hdac3. Moreover, using a Hippo pathway inhibitor and overexpressing the transcriptional regulator Yap (Yes-associated protein) significantly reduced *Hamp* mRNA levels. Using a promoter reporter assay, we then identified 2 Yap-binding repressor sites within the human *HAMP* promoter region. We also found that inhibiting Hdac3 led to increased translocation of Yap to the nucleus, suggesting activation of Yap. Notably, knock-in mice expressing a constitutively active form of Yap (Yap K342M) phenocopied the altered hepcidin levels observed in *Hdac3*-LKO mice. Mechanistically, we show that iron-overload-induced ferroptosis underlies the liver injury that develops in *Hdac3*-LKO mice, and knocking down Yap expression in *Hdac3*-LKO mice reduces both iron-overload- and ferroptosis-induced liver injury. These results provide compelling evidence supporting the notion that HDAC3 regulates iron homeostasis via the Hippo/Yap pathway and may serve as a target for reducing ferroptosis in iron-overload-related diseases.

## Introduction

Iron is an essential element in the human body, playing a critical role in regulating various physiological processes. In the body, iron homeostasis is regulated by the coordinated absorption, utilization, storage, and recycling of iron [1]. Interestingly, both iron deficiency and iron overload can lead to a variety of pathological conditions. For example, excess iron can cause progressive and even irreversible tissue damage. However, the precise mechanisms that regulate iron homeostasis in response to iron overload remain poorly understood.

In mammals, systemic iron homeostasis is tightly regulated by the hepcidin–FPN (ferroportin, also known as SLC40A1) axis [2]. As the central regulator of iron metabolism, the peptide hormone hepcidin (encoded by the *HAMP* gene) mediates the internalization and degradation of FPN, leading to decreased iron absorption in the duodenum and decreased iron recycling in the reticuloendothelial system [3]. The canonical pathways that regulate hepcidin levels include the bone morphogenetic proteins (BMPs)/Hemojuvelin (HJV)/small mothers against decapentaplegic (SMAD) signaling pathways [4,5] and the interleukin-6 (IL-6)/signal transducer and activator of transcription 3 (STAT3) inflammation signaling pathway [6]. Iron loading increases BMP6 expression, which—together with HJV—activates type 1 (e.g., ALK2 and ALK3) and type 2 (e.g., BMPR2 and ACVR2A) BMP serine-threonine kinase receptors, leading to phosphorylation of receptor-activated SMAD proteins and the formation of active transcriptional complexes with SMAD4 [7]. In contrast, matriptase-2 (encoded by the *TMPRSS6*[Transmembrane Serine Protease 6] gene), a type II transmembrane serine protease that cleaves HJV to produce a soluble form of HJV (sHJV), suppresses BMP/SMAD signaling [8]. During inflammation, IL-6 binds to the IL-6 receptor, activating Janus kinase (JAK) tyrosine kinases, which, in turn, triggers the formation of STAT3 complexes that bind to the *HAMP* promoter in the nucleus. *HAMP* expression can also be stimulated by the cytokine activin B, a process that is also dependent on the BMP/SMAD signaling pathway [1].

Recently, non-canonical *HAMP* regulators—in particular, epigenetic modulators—have drawn considerable attention. For example, modulators of epigenetic changes such as acetylation have been suggested to regulate iron metabolism. Yang et al. [9] identified the nicotinamide adenine dinucleotide (NAD)-dependent protein deacetylase Sirtuin-2 (SIRT2) as a key regulator of FPN expression. Moreover, during cerebral ischemia, the transcription factor NF-κB undergoes acetylation of the RelA subunit at Lys310, thereby upregulating expression of the divalent metal transporter DMT1, leading to increased iron content in the brain and subsequent damage to post-ischemic neurons [10]. In addition, in vitro experiments showed that histone deacetylase (HDAC) inhibitors can upregulate the expression of ferritin H (FTH) by promoting binding of the transcription factor Sp1 to the FTH promoter without affecting acetylation levels [11]. In a prior study, we reported that the methyl-CpG-binding protein MBD5 governs the expression of Fth by modulating histone acetylation [12]. This observation implies a linkage between alterations in epigenetic marks and the regulation of iron homeostasis. Interestingly, reduced *HAMP* expression—accompanied by highly methylated CpG island sites within the *HAMP* promoter region—has been observed in patients with liver cancer [13]. Notably, *HAMP* expression is regulated primarily by an increase in histone acetylation at its promoter region, although in vitro experiments have shown that *SMAD4* overexpression induces hyperacetylation of histone H3K9 in the *HAMP* promoter region, resulting in transcriptional upregulation of *HAMP* [14]. In addition, HDAC inhibitors have been shown to upregulate *HAMP* expression in HepG2 cells, a human liver cancer cell line [15]. Nevertheless, additional studies are needed in order to determine whether—and if so, how—*HAMP* expression is regulated by HDACs under physiological conditions.

The Hippo signaling pathway, also known as the Salvador/Warts/Hippo pathway, is termed by the protein kinase Hippo in *Drosophila* [16]. Specific gene deletion of the upstream Hippo kinases in the liver, such as MST1 and MST2 [17] and LATS1 and LATS2 [18], or their scaffold proteins SAV1 [19] and MOB1A and MOB1B [20], could lead to decreased phosphorylation of yes-associated protein (YAP) and Tafazzin (TAZ), followed by their translocation into the nucleus, which then binds to TEA domain transcription factors (TEADs) or other transcription factors to trigger a series of proliferative and anti-apoptotic gene expression, causing hepatomegaly and liver cancer [16,18–20]. However, whether the Hippo signaling pathway regulates *HAMP* expression is largely unknown.

We previously reported that HDAC1 is a novel suppressor of *HAMP* expression by competitively binding to SMAD4 via a process not dependent on BMP/SMAD1/5/8 signaling [21]. Interestingly, we also found that the HDAC3 inhibitor RGFP966 had no effect on *HAMP* expression in vitro [21], although another study found that RGFP966 increases *HAMP* expression in iron-deficient mice [22]. Here, we investigated whether HDAC3 plays a role in regulating iron homeostasis and mediating ferroptosis in vivo dependent on the Hippo signaling pathway.

## Results

### Hepatocyte-specific *Hdac3*-deficient mice have impaired iron metabolism

**Fig. 1.**
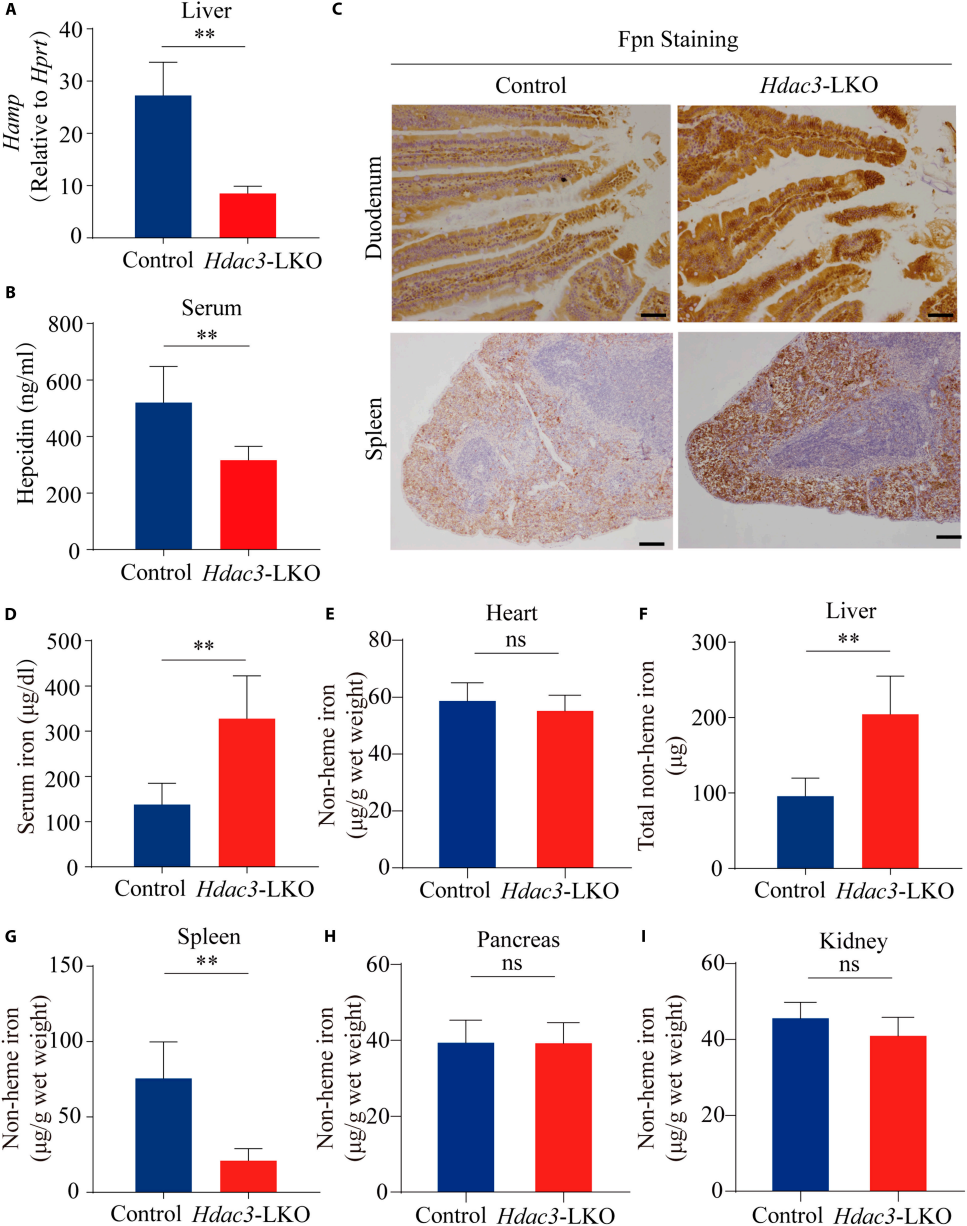
Liver-specific *Hdac3* knockout mice (*Hdac3*-LKO mice) develop iron overload via the hepcidin–ferroportin axis. (A and B) Hepatic *Hamp* mRNA (A) and serum hepcidin levels were measured in 8-week-old *Hdac3*-LKO mice and control littermates. (C) Duodenum (top) and spleen (bottom) sections were prepared from the indicated mice and stained for Fpn. Scale bar = 100 μm. (D to I) Summary of serum iron (D), and non-heme iron measured in the indicated tissues (E to I) (*n* ≥ 6 mice per group). **P < 0.01 and ns, not significant (Student’s *t* test).

To examine the role of HDAC3 in systemic iron homeostasis, we generated mice lacking *Hdac3* expression selectively in the liver (referred to hereafter as *Hdac3*-LKO mice) by crossing *Hdac3*^fl/fl^ mice with Alb-Cre^+^ transgenic mice. We found that compared to control littermates, *Hdac3*-LKO mice develop hepatic lesions (Fig. S1A) and have an increased liver/body weight ratio (Fig. S1B), consistent with previous reports [23]. As expected, *Hdac3*-LKO mice have significantly reduced *Hamp* mRNA levels in the liver (Fig. 1A), as well as reduced serum hepcidin levels (Fig. 1B) when fed a standard-iron diet, with an accumulation of Fpn protein in the duodenum and spleen (Fig. 1C) compared to control mice, indicating that *Hdac3*-LKO mice have increased intestinal iron absorption and increased splenic iron recycling. Moreover, *Hdac3*-LKO mice have higher serum iron levels compared to controls (Fig. 1D). We found no significant difference between *Hdac3*-LKO and control mice with respect to non-heme iron content in the heart (Fig. 1E), pancreas (Fig. 1H), or kidneys (Fig. 1I); in contrast, *Hdac3*-LKO mice had significantly higher levels of total non-heme iron in the liver (204.38 μg) compared with control mice (95.75 μg) (Fig. 1F), suggesting increased hepatic iron storage, and significantly lower splenic non-heme iron content (Fig. 1G). Moreover, *Hdac3*-LKO mice fed a high-iron diet (HID) had reduced hepatic *Hamp* mRNA levels (Fig. S2A), higher serum iron levels (Fig. S2B), higher hepatic total non-heme iron levels (1,872.04 μg vs. 877.10 μg) (Fig. S2C), lower splenic iron levels (Fig. S2D), and higher non-heme iron levels in the kidney, pancreas, and heart (Fig. S2E) compared to HID-fed control mice. Together, these findings suggest that hepatic HDAC3 plays an essential role in regulating hepatic *HAMP* expression and systemic iron homeostasis.

### HDAC3 regulates HAMP expression via the YAP signaling pathway

HDAC3 is the only class I deacetylase that can interact with acetylated H3K9 (H3K9ac) [23]. Moreover, mice lacking hepatic *Hdac3* have significantly increased levels of H3K9ac [24]. To determine whether Hdac3 directly regulates *Hamp* expression, we measured H3K9ac levels at the *Hamp* promoter. We found that *Hdac3*-LKO mice have significantly reduced levels of H3K9ac at the *Hamp* promoter region (Fig. S3A), but significantly higher levels of H3K9ac at the promoter region of the Hdac3-targeted *p21* gene (Fig. S3B) compared to control mice, suggesting that the *HAMP* gene is not a direct target gene of HDAC3.

**Fig. 2.**
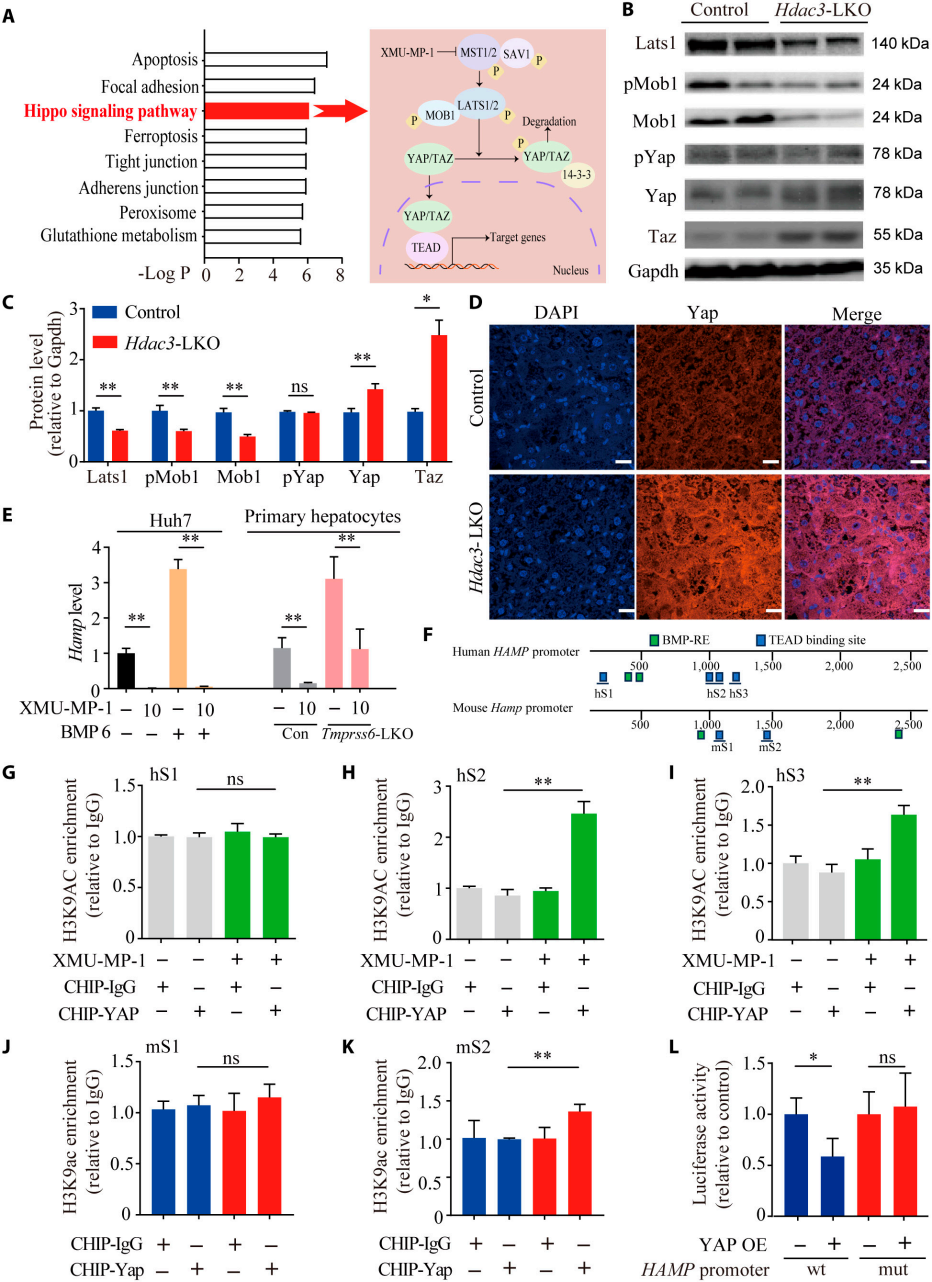
HDAC3 regulates *HAMP* expression by regulating YAP transcriptional activity. (A) Left: KEGG enrichment of hepatic transcription analysis of *Hdac3*-LKO mice (GSE22457). Right: the diagram of the Hippo signaling pathway. (B and C) Western blot analysis (B) and quantification (C) of the indicated proteins in the Hippo pathway measured in *Hdac3*-LKO and control mice. Four replicates were done for the blots. (D) Liver sections were prepared from *Hdac3*-LKO and control mice and immunostained for Yap; the nuclei were counterstained with DAPI. Scale bar = 100 μm. (E) Left, summary of relative *HAMP* mRNA levels measured in Huh7 cells treated with 10 μM XMU-MP-1 or vehicle in the presence or absence of BMP6; right, summary of relative *Hamp* mRNA levels measured in primary hepatocytes prepared from *Tmprss6*-LKO and control mice treated with 10 μM XMU-MP-1 or vehicle. (F) Schematic diagram depicting the human *HAMP* promoter and the mouse *Hamp* promoter. The approximate locations of BMP-responsive elements (BMP-RE) and TEAD-binding sites are indicated. (G to I) YAP ChIP was performed using Huh7 cells treated with 10 μM XMU-MP-1 or vehicle followed by quantitative RT-PCR of the *hHAMP* promoter to amplify the TEAD-binding sites hS1 (G), hS2 (H), and hS3 (I). (J and K) YAP ChIP was performed using hepatocytes prepared from *Hdac3*-LKO (red bars) and control mice (blue bars) followed by quantitative RT-PCR of the *mHamp* promoter to amplify the TEAD-binding sites mS1 (J) and mS2 (K). (L) Relative luciferase activity using the *hHAMP* promoter or *hHAMP-MUT* promoter was measured in Huh7 cells overexpressing YAP (YAP OE). *P < 0.05, **P < 0.01, and ns, not significant (Student’s *t* test).

We further asked whether canonical regulatory pathways play a role in regulating *HAMP* gene expression. We found that the levels of both p-Stat3 and p-Smad1/5 were significantly higher in *Hdac3*-LKO mice compared to control mice (Fig. S3C and D), but these results could not explain the reduced *Hamp* expression measured in *Hdac3*-LKO mice, suggesting that other pathways are likely involved in regulating *Hamp* expression. We therefore used the GEO (Gene Expression Omnibus) database to examine hepatic transcription profiling of *Hdac3*-LKO mice (GSE22457) [25]. KEGG (Kyoto Encyclopedia of Genes and Genomes) enrichment analysis revealed that several pathways and processes were affected in the *Hdac3*-LKO mice, including apoptosis, focal adhesion, the Hippo pathway, ferroptosis, and glutathione metabolism (Fig. 2A). In addition, we found that the *Cdh1*, *Bmp8b*, *Ajuba*, *Ccnd1*, *Tead1*, and *Bmp6* genes were upregulated, while the *Fzd8* and *Fgf1* genes were downregulated, among the altered genes involved in the Hippo pathway (Fig. S3E); thus, the reported phenotype of hepatomegaly and liver tumorigenesis observed in *Hdac3*-LKO mice may be explained by altered regulation of the Hippo pathway [17,26,27].

YAP (a major component in the Hippo pathway) has been shown to regulate expression of the transferrin receptor 1 (TfR1) [28], a key component in iron metabolism, suggesting that YAP may be involved in regulating iron metabolism. We therefore examined whether the Hippo pathway plays a role in regulating *HAMP* expression. We found that compared to control mice, *Hdac3*-LKO mice have significantly reduced protein levels of Lats1, p-Mob1, and Mob1 (Fig. 2B and C), suggesting reduced Hippo pathway activity. In contrast, we found significantly higher levels of both total Yap protein and Taz protein in the livers of *Hdac3*-LKO mice, with no difference in p-Yap levels (Fig. 2B and C). Confocal imaging of liver sections immunostained for Yap confirmed higher expression of hepatic Yap in *Hdac3*-LKO mice compared to control mice, indicating increased nuclear localization of Yap (Fig. 2D). In addition, treating Huh7 cells (a hepatocyte-derived carcinoma cell line) with the HDAC3 inhibitor RGFP966 increased YAP immunofluorescence in the nucleus (Fig. S4A and B), and Western blot analysis confirmed that RGFP966 decreased YAP in the cytoplasm and increased YAP in the nucleus (Fig. S4C to F). Taken together, these data suggest that inhibition of hepatic HDAC3—either genetically or pharmacologically—could reduce the activation of the Hippo pathway. To examine further whether the Hippo pathway regulates *HAMP* expression, we treated Huh7 cells with XMU-MP-1, which inhibits the Hippo pathway kinases MST1 and MST2 [29]. We found that *HAMP* expression was significantly reduced in Huh7 cells following XMU-MP-1 treatment, even in the presence of BMP6 (Fig. 2E, left). To confirm that XMU-MP-1 treatment leads to reduced *Hamp* expression, we used primary hepatocytes isolated from *Tmprss6*-LKO mice, which lack transmembrane serine protease 6 expression in the liver [30]. We found that XMU-MP-1 significantly reduced *Hamp* expression in primary hepatocytes from both *Tmprss6*-LKO mice and control littermates (Fig. 2E, right). Moreover, overexpressing *YAP* and *TAZ* in Huh7 cells significantly reduced *HAMP* expression, whereas overexpressing either *TEAD1* or *TEAD4* had no effect on *HAMP* expression (Fig. S4G). Taken together, these results suggest that reduced Hippo pathway activity may underlie the reduced *Hamp* expression observed in *Hdac3*-LKO mice.

### YAP regulates *HAMP* expression by binding directly to TEADs

As a transcriptional co-activator, YAP can either promote [31] or inhibit [32–34] target gene transcription, depending on a variety of mechanisms. To examine the mechanism by which YAP regulates *HAMP* expression, we performed co-immunoprecipitation (co-IP) in Huh7 cell lysates to test whether YAP interacts with the 3 principal transcription factors that regulate *HAMP* expression—namely, SMAD4, SMAD1/5, and STAT3—and found no apparent interaction (Fig. S5A and B); as a positive control, we confirmed that TAZ interacts with YAP (Fig. S5A). This result suggests that YAP regulates *HAMP* via a mechanism independent of SMAD4, SMAD1/5, and STAT3. We therefore hypothesized that YAP may drive transcription of the *HAMP* promoter via a more direct mechanism. For example, YAP can form a complex with TEAD1 to TEAD4, enabling TEAD1 to TEAD4 to recognize consensus binding sites (GGAATG) in the genome, thereby exerting transcriptional regulation [32]. Notably, we found this TEAD-binding motif within both the human *HAMP* promoter and the mouse *Hamp* promoter (Fig. 2F).

To determine whether YAP interacts with the *HAMP* promoter, we treated Huh7 cells with XMU-MP-1 and then performed chromatin immunoprecipitation (ChIP)-PCR analysis. Compared to untreated cells, cells treated with XMU-MP-1 had higher levels of YAP binding at the human *HAMP* promoter at 2 specific sites, hS2 and hS3 (Fig. 2G to I). Consistent with these results, we found increased Yap binding at the mS2 site in mouse *Hamp* promoter in the liver of *Hdac3*-LKO mice compared to control mice (Fig. 2J and K). These findings suggest that *HAMP* is a newly identified target gene for YAP binding. In addition, overexpressing YAP in Huh7 cells significantly downregulated activity of the wild-type *HAMP* promoter measured using a luciferase reporter assay (Fig. 2L), but had no effect in cells expressing a *HAMP* promoter containing mutations in the hS2 and hS3 sites (Fig. 2L). Importantly, as noted above, histone acetylation at the *Hamp* promoter region was significantly reduced in the liver of *Hdac3*-LKO mice compared to control mice (Fig. S3A), consistent with a previous study showing that YAP represses transcriptional activity by directly reducing histone acetylation at the target gene’s promoter region [32]. Taken together, these findings suggest that proteins in the Hippo pathway transcriptionally regulate *HAMP* expression via direct binding to its promoter region.

### Yap K342M knock-in mice have reduced *Hamp* expression

**Fig. 3.**
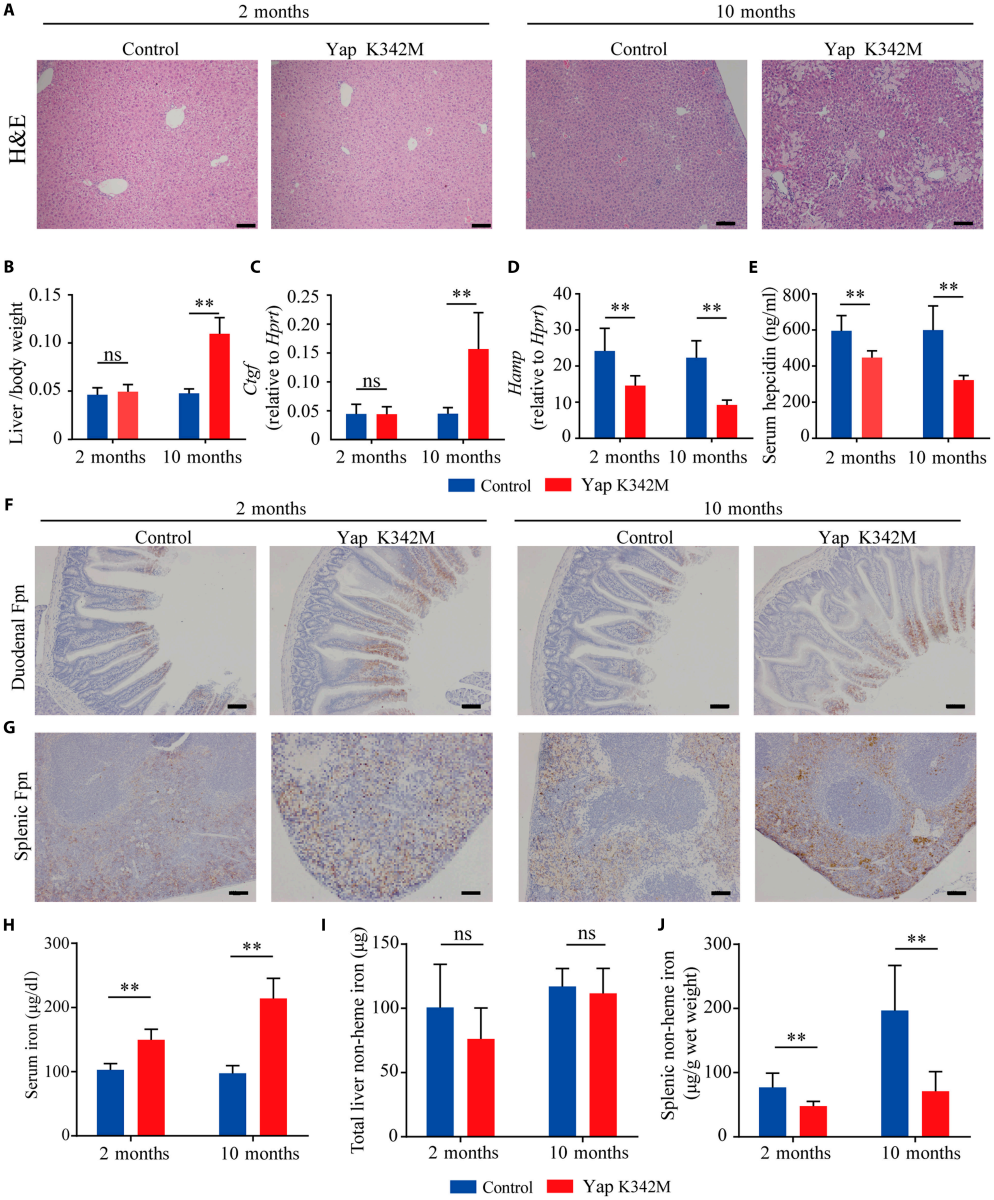
Knock-in mice expressing a constitutively active form of Yap showed decreased hepcidin and increased serum iron levels. (A) Liver sections were prepared from 2- and 10-month-old Yap K342M and control mice and stained with H&E. (B to E) Summary of the liver/body weight ratio (B), Hepatic *Ctgf* mRNA levels (C), hepatic *Hamp* mRNA levels (D), and serum hepcidin levels (E) in the indicated mice at the indicated ages; *n* ≥ 6 mice per group. (F and G) Duodenum (F) and spleen (G) sections were prepared from 2- and 10-month-old Yap K342M and control mice and stained for Fpn. (H to J) Summary of serum iron (H), hepatic non-heme iron (I), and splenic non-heme iron (J); *n* ≥ 6 mice per group. Scale bar = 100 μm. **P < 0.01 and ns, not significant (Student’s *t* test).

Next, we examined the role of YAP in regulating *HAMP* expression using a knock-in mouse that expresses a constitutively active form of Yap containing a Lys>Met mutation at residue 342 (referred to hereafter as Yap K342M mice) [35]. At 2 months of age, we found no significant difference in liver histology (based on hematoxylin and eosin [H&E] staining), the liver/body weight ratio, or expression of the Yap target gene *Ctgf* between Yap K342M mice and control mice (Fig. 3A to C). However, at 10 months of age, the Yap K342M mice develop visible liver lesions (Fig. 3A), a significantly higher liver/body weight ratio (Fig. 3B), and higher expression of *Ctgf* (Fig. 3C) compared to age-matched controls. Interestingly, we found decreased levels of hepatic *Hamp* mRNA (Fig. 3D) and serum hepcidin protein (Fig. 3E) in Yap K342M mice at both 2 and 10 months of age compared to age-matched controls. Consistent with this finding, at both 2 and 10 months, the Yap K342M mice had altered iron homeostasis compared to control mice, as evidenced by an accumulation of Fpn in the duodenum (Fig. 3F) and spleen (Fig. 3G), as well as higher serum iron levels (Fig. 3H). Although we found no difference in total hepatic iron content at either 2 or 10 months of age (Fig. 3I), splenic iron content was lower in both 2- and 10-month-old Yap K342M mice compared to age-matched controls (Fig. 3J). Thus, Yap K342M mice have a phenotype that resembles *Hdac3*-LKO mice, suggesting that YAP serves as a key factor downstream of HDAC3 in regulating *HAMP* expression and systemic iron homeostasis.

### *Hdac3*-LKO mice have increased liver damage mediated by iron-overload-induced ferroptosis

**Fig. 4.**
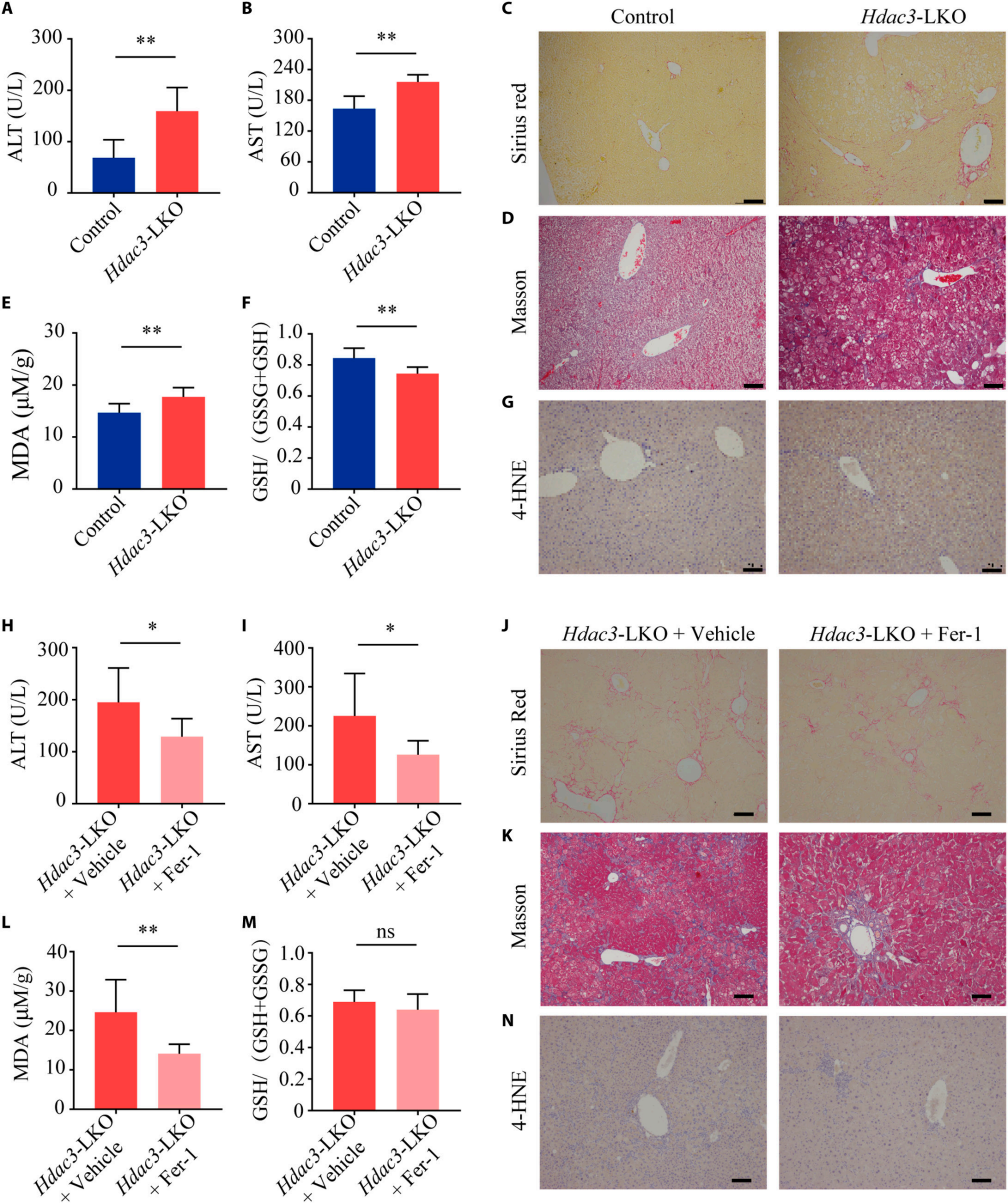
Ferroptosis plays a role in liver injury in *Hdac3*-LKO mice. (A, B, E, and F) Summary of serum ALT (A), serum AST (B), hepatic MDA (E), and hepatic GSH/(GSH+GSSG) ratio (F) measured in 8-week-old *Hdac3*-LKO and control mice; *n* ≥ 6 mice per group. (C, D, and G) Liver sections were prepared from 8-week-old *Hdac3*-LKO and control mice and stained with Sirius red (C), Masson’s trichrome (D), and 4-HNE antibody (G). (H, I, L, and M) Summary of serum ALT (H), serum AST (I), hepatic MDA (L), and hepatic GSH/(GSH+GSSG) ratio (M) measured in *Hdac3*-LKO mice treated with Fer-1 or vehicle; *n* ≥ 6 mice per group. (J, K, and N) Liver sections were prepared from vehicle and Fer-1-treated *Hdac3*-LKO mice and stained with Sirius red (J), Masson’s trichrome (K), and 4-HNE antibody (N). Scale bar = 100 μm. *P < 0.05, **P < 0.01, and ns, not significant (Student’s *t* test).

As noted above, we found that our *Hdac3*-LKO mice have significantly enlarged livers compared to controls. We therefore examined whether these mice also have increased liver injury. Indeed, we found that *Hdac3*-LKO mice have increased serum alanine transaminase (ALT) and aspartate transaminase (AST) levels (Fig. 4A and B), as well as increased Sirius red and Masson’s trichrome staining of liver sections, indicating liver fibrosis (Fig. 4C and D).

Previous studies found that iron-overload-induced liver fibrosis is mediated by ferroptosis, an iron-dependent form of cell death [36–41]. Moreover, we found that the ferroptosis pathway was affected in *Hdac3*-LKO mice (see Fig. 2A), and these mice develop iron overload in the liver (see Fig.21F). Consequently, we assessed several potential biomarkers of ferroptosis and observed a slight but statistically significant increase in hepatic MDA (malondialdehyde) levels (Fig. 4E), while the hepatic glutathione (GSH)/(GSH+oxidized glutathione [GSSG]) ratio was slightly—albeit significantly—decreased (Fig. 4F) in *Hdac3*-LKO mice compared to control mice. In addition, liver sections from *Hdac3*-LKO mice had stronger staining for the lipid peroxidation product 4-hydroxynonenal (4-HNE) compared to control mice (Fig. 4G). Taken together, these results suggest that the loss of hepatic *Hdac3* leads to liver fibrosis, potentially via ferroptosis.

To test this hypothesis, we treated *Hdac3*-LKO mice with ferrostatin-1 (Fer-1), which selectively blocks ferroptosis by inhibiting lipid peroxidation and preventing damage to lipid membranes [36,42] .We found that compared to vehicle-treated *Hdac3*-LKO mice, Fer-1–treated *Hdac3*-LKO mice had significantly lower levels of serum ALT (Fig. 4H) and AST (Fig. 4I) as well as reduced Sirius red (Fig. 4J) and Masson’s trichrome (Fig. 4K) staining of liver sections, indicating that Fer-1 treatment reduces liver fibrosis in *Hdac3*-LKO mice. Moreover, Fer-1 treatment significantly decreased hepatic MDA levels (Fig. 4L) and prevented the decrease in the GSH/(GSH+GSSG) ratio (Fig. 4M) and the increase in 4-HNE staining (Fig. 4N) in the liver of *Hdac3*-LKO mice. Thus, inhibiting ferroptosis reduces liver fibrosis in *Hdac3*-LKO mice.

### Knocking down Yap significantly reduces iron overload and ferroptosis-mediated liver injury in *Hdac3*-LKO mice

**Fig. 5.**
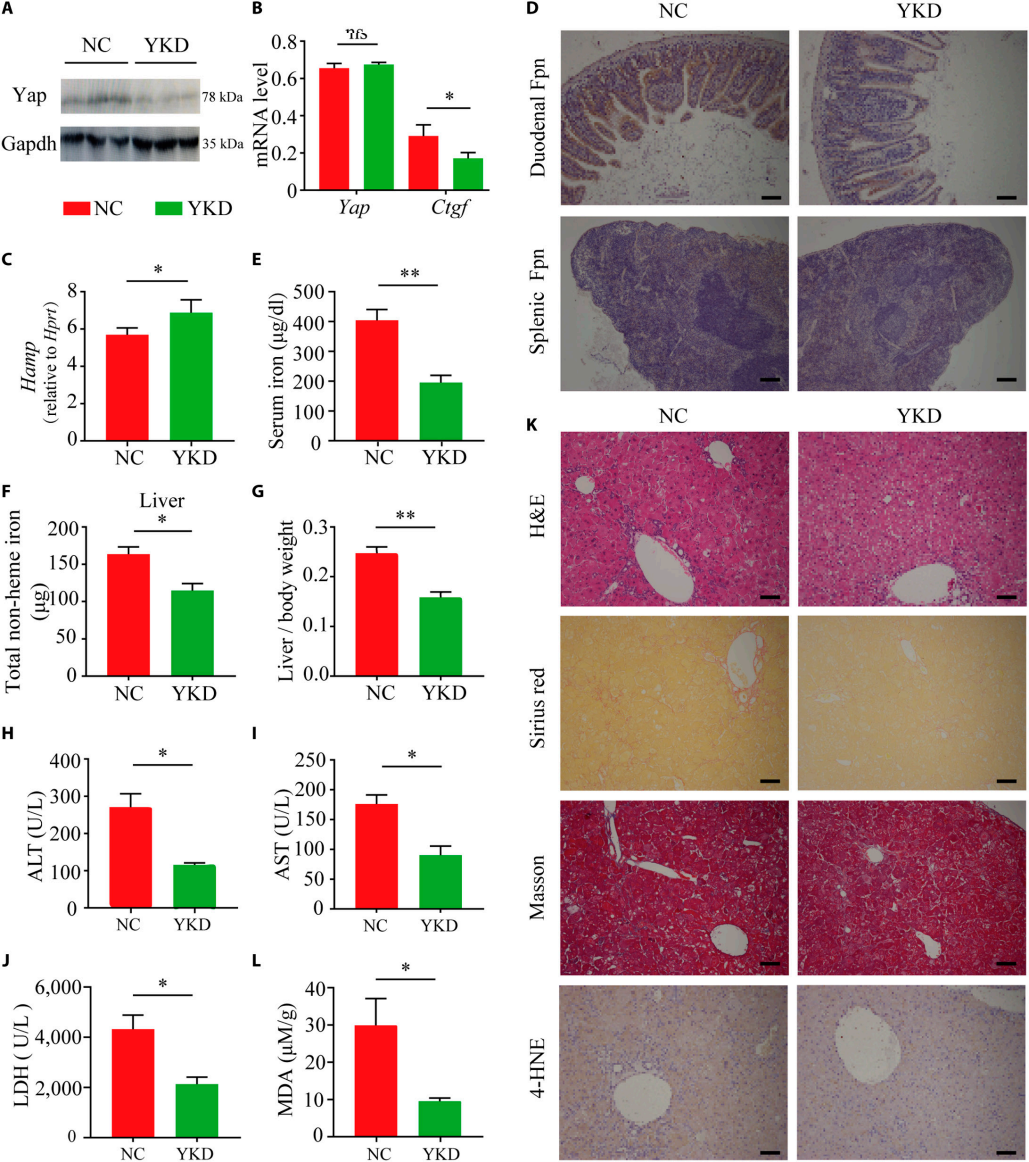
Knocking down *Yap* in *Hdac3*-LKO mice reduces iron overload and ferroptosis-induced liver injury. At 5 weeks of age, *Hdac3*-LKO mice received an intravenous injection of pAAV-TBG-*shYap* or pAAV-TBG-NC (10^11^ vg/g); 4 weeks later, the mice were sacrificed and analyzed. (A) Western blot analysis of hepatic *Yap* protein. (B, C, E to J, and L) Summary of hepatic *Yap* mRNA and *Ctgf* mRNA (B), hepatic *Hamp* mRNA (C), serum iron (E), hepatic non-heme iron (F), liver/body weight ratio (G), serum ALT (H), serum AST (I), serum LDH (J), and hepatic MDA (L) measured in the indicated mice. (D) Duodenum and spleen sections were prepared from the indicated mice and stained for Fpn. Scale bar = 100 μm. (K) Liver sections were prepared from the indicated mice and stained with H&E, Sirius red, Mason’s trichrome, and 4-HNE antibody. *n* ≥ 6 mice per group. Scale bar = 100 μm. *P < 0.05, **P < 0.01, and ns, not significant (Student’s *t* test).

Lastly, we examined whether Yap plays a major role in both iron overload and ferroptosis-mediated liver injury in *Hdac3*-LKO mice. To systemically knock down *Yap*, we gave 5-week-old *Hdac3*-LKO mice an intravenous injection of a hepatocyte-specific targeting adeno-associated virus (TBG-AAV) expressing sh*Yap* (YKD) or a negative control (NC) AAV; 4 weeks after injection, the mice were sacrificed and analyzed. We found that *Hdac3*-LKO mice expressing the YKD AAV had reduced hepatic Yap protein compared to mice expressing the control NC AAV (Fig. 5A), despite no difference in hepatic *Yap* mRNA levels (Fig. 5B); moreover, knocking down *Yap* reduced the level of *Ctgf* mRNA (a Yap target gene) (Fig. 5B), suggesting that the translation of *Yap* mRNA and Yap’s downstream targets were successfully blocked by expressing the YKD AAV. Importantly, knocking down *Yap* in *Hdac3*-LKO mice significantly increased hepatic *Hamp* expression (Fig. 5C) and reduced Fpn protein levels in both the duodenum and spleen (Fig. 5D) compared to NC-treated *Hdac3*-LKO mice. Consistent with these changes, knocking down *Yap* also reduced serum iron levels (Fig. 5E) and hepatic non-heme iron content (Fig. 5F), but had no effect on iron content measured in the spleen, kidney, pancreas, intestine, or heart (Fig. S6). In addition, knocking down *Yap* in *Hdac3*-LKO mice significantly reduced the liver/body weight ratio (Fig. 5G), as well as serum ALT, AST, and lactate dehydrogenase (LDH) levels (Fig. 5H to J). Moreover, liver fibrosis was reduced based on H&E, Sirius red, and Masson’s trichrome staining (Fig. 5K). Finally, knocking down *Yap* reduced 4-HNE staining in liver sections (Fig. 5K) and significantly reduced hepatic MDA levels (Fig. 5L) compared to NC-treated *Hdac3*-LKO mice. In conclusion, these findings collectively suggest that targeting YAP could represent a promising therapeutic approach for mitigating liver damage induced by ferroptosis, particularly in the context of reduced HDAC3.

## Discussion

Here, we show that loss of hepatic *Hdac3* leads to hepatic iron overload via Yap-mediated suppression of *Hamp* expression. Moreover, we show that hepatic *Hdac3* exerts its protective role in the liver primarily by maintaining systemic iron homeostasis and reducing ferroptosis. Importantly, we also show that knocking down *Yap* expression significantly reduces iron accumulation in the liver by upregulating *Hamp* expression, and can reduce liver injury in *Hdac3*-LKO mice by inhibiting ferroptosis.

Although HDAC family members have been suggested to modulate *HAMP* expression, the results are somewhat inconclusive. For example, the class I and II HDAC inhibitor trichostatin A has been shown to increase *HAMP* expression in both in vitro and in mouse models [43,44], and the non-selective HDAC inhibitor suberoylanilide hydroxamic acid has been shown to upregulate *HAMP* expression by inhibiting HDAC3 in both Huh7 cells and mouse primary hepatocytes [45]. In addition, knocking down HDAC3 has also been shown to upregulate *HAMP* expression in Huh7 cells [22]. In iron-deficient mice, the HDAC3 inhibitor RGFP966 has been shown to increase *Hamp* expression [22]. However, this pharmacological approach might not be Hdac3 specific, raising concerns about potential off-target effects. By contrast, our study applied liver-specific genetic knockout approach, which is more specific to the targeted gene *Hdac3* in the liver. To the best of our knowledge, our study is the first to functionally demonstrate that mice lacking hepatic Hdac3 have significantly reduced *Hamp* expression, with subsequent iron accumulation in several tissues, particularly the liver.

Altered Hippo signaling has been well documented as causing various liver diseases such as hepatomegaly and hepatocellular carcinoma [26,27]. However, whether Hippo signaling plays a role in maintaining iron homeostasis remains an open question. A recent study suggests that YAP—as a downstream effector molecule of the Hippo pathway—may modulate TfR1 [46]. Interestingly, deleting Warts (Lats1/2 in mammals) in *Drosophila* led to overexpression of *Fer1HCH*, the Drosophila homolog of human FTH1 [28,46]. Here, we report that YAP is a novel regulator of *HAMP* expression, and we show that the Hippo-YAP axis may be targeted in order to modulate iron metabolism by directly increasing *HAMP* expression. In Yap K342M mice, it is intriguing that the hepatic iron levels remain unchanged despite having decreased hepcidin levels and increased serum iron levels compared with controls. One possibility is the potential extra-hepatic effect of YAP on hepatic iron levels as we used global Yap K342M mice. The other possibility is the potential effect of Yap on other iron transporters, such as TfR1, Slc39a14, DMT1, and Fpn.

Due to the Fenton reaction, excessive iron directly leads to detrimental effects in various tissues by activating free radicals and ROS, inducing DNA damage and causing degradation of cellular components [47,48]. Moreover, a growing number of studies highlight the pathological role that iron-dependent cell death (i.e., ferroptosis) plays in liver disease [36,37,42,49–52]. Using various genetic mouse models, our group and others previously showed that ferroptosis plays an important role in iron-overload-induced liver injury and fibrosis, and inhibiting ferroptosis—for example, using approaches such as Fer-1 treatment, knocking out hepatic *Slc39a14*, and overexpressing *FGF21* (fibroblast growth factor 21) and *PPARs* (peroxisome proliferator-activated receptors)—has been shown to ameliorate iron-overload-induced liver disease [36,37,53,54]. Consistent with our findings, both the Hippo pathway [17,45,54,55] and knocking out *HDAC3* [23,24,56] have been associated with ferroptosis. Here, we report that knocking out *dac3* in hepatocytes triggers ferroptosis via a novel mechanism, namely, Yap-mediated suppression of *Hamp* expression. Although we demonstrate that ferroptosis serves as one of the pathogenic mechanisms that account for driving liver injury in *Hdac3*-LKO mice, there might exist additional mechanisms. Nevertheless, future studies are needed to explore the precise underlying mechanism.

A wealth of evidence indicates that HDAC3 inhibitors such as RGFP966, MI-192, and BG45 may hold promise for treating various diseases, including cancer [57]. Thus, the emergence of new, more potent and selective HDAC3 inhibitors may provide new therapeutic options for treating various cancer types while minimizing the risk of adverse effects. In this respect, our findings shed new light on the potential detrimental effects of HDAC3 inhibition in a clinical setting, including altered iron homeostasis and the subsequent ferroptosis-induced liver injury. Given that several components of the Hippo pathway exert tumor-suppressive properties [26,27], and given that altered YAP/TAZ activity has been implicated in various diseases [29,34], therapeutic interventions designed to target this pathway hold significant promise for treating these diseases. Indeed, several compounds that disrupt YAP/TAZ-TEAD interactions were shown to have anti-tumor effects in various animal models of cancer [58]. From the perspective of targeting the Hippo pathway, our study suggests a feasible strategy for the prevention and treatment of tissue injury.

In summary, our study provides compelling evidence that dac3 serves as a novel epigenetic suppressor of *Hamp* expression. Using a hepatocyte-specific knockout mouse model, we show that loss of *Hdac3* inhibits the Hippo pathway, leading to increased Yap activity, ultimately suppressing *Hamp* transcription. Furthermore, we show that the loss of hepatic *Hdac3* leads to ferroptosis, thereby causing liver damage. Importantly, we also show that knocking down *Yap* in *Hdac3*-LKO mice ameliorates both iron overload and ferroptosis-induced liver injury. These findings suggest that targeting HDAC3 and/or the Hippo pathway may serve as a novel therapeutic strategy for the treatment of iron overload and/or ferroptosis-related diseases.

## Materials and Methods

### Animal experiments

All animal experiments were conducted in accordance with the guidelines and regulations approved by the Institutional Animal Care and Use Committee of Zhejiang University. C57BL/6 mice were procured from SLAC Laboratory Animal Co., Ltd. (Shanghai, China) and were maintained in a specific pathogen-free environment and (except where indicated otherwise) fed an egg-white-based diet (AIN-76A; Research Diets, Inc., New Brunswick, NJ) containing 50 mg/kg iron. Mice of various strains were assigned randomly to receive the indicated treatments. Where indicated, *Hdac3*-LKO and littermate controls were given an intraperitoneal injection of vehicle or Fer-1 (Selleck) (1 mg/kg body weight) every other day for 3 weeks [36]. To knock down *Yap*, 5-week-old mice received an intravenous injection of the TBG-AAV expressing sh*Yap* (pAAV-TBG-sh*Yap*; YKD) or a non-silencing negative control (pAAV-TBG-NC; NC) (Obio Technology, Shanghai, China) via tail vein injection at 10^11^ viral genome copies/g body weight; the mice were sacrificed at 9 weeks of age, and tissue samples were gathered for subsequent analysis.

### Cell culture and treatment

HEK293T and Huh7 cells were purchased from the Shanghai Cell Bank and were cultured in Dulbecco’s modified Eagle’s medium (Gibco), supplemented with 10% (v/v) FBS (fetal bovine serum, Gibco) and 1× penicillin–streptomycin (Gibco). The cells were maintained at 37 °C in a humidified atmosphere with 5% CO_2_. For XMU-MP-1 treatment, Huh7 cells were seeded in six-well plates and cultured with either 0.1% dimethyl sulfoxide (DMSO; as a control) or XMU-MP-1 (MedChemExpress) for 12 h. Following this, the cells were harvested for mRNA and Western blot analysis. Primary hepatocytes were isolated from 8-week-old male *Tmprss6*-LKO and control mice using the previously described collagenase isolation method [30,37].

### Luciferase reporter assay

The reporter plasmid consisted of pGL3-*HAMP*, encompassing the 2.7-kb 5′-flanking genomic region of the human *HAMP* gene and the 5′-UTR (ranging from −2,700 to +71 bp), as well as the pGL3-*HAMP*-*mutation* plasmid, derived from pGL3-*HAMP*, featuring mutated TEAD-binding sites. Huh7 cells, cultivated in 24-well plates at a density of 10^5^ cells per well, underwent transient transfection with 490 ng per well of either the pGL3-*HAMP* or the pGL3-*HAMP-mutation* plasmid, in conjunction with 10 ng per well of the Renilla luciferase plasmid using the FuGENE HD Transfection Reagent (Roche Applied Sciences, Indianapolis, IN) [21]. Thirty-six hours after transfection, either 0.1% DMSO (vehicle) or XMU-MP-1 was added to the wells, and the cells were cultured for an additional 12 h. Luciferase activity analysis was conducted using the Dual-Luciferase Reporter Assay System (Promega) in accordance with the manufacturer’s instructions, and were normalized to Renilla luminescence [59].

### RNA isolation and quantitative real-time PCR

Total RNA was extracted, and quantitative real-time PCR (RT-PCR) was conducted as previously outlined [21], utilizing the primers detailed in Table S1.

### Western blot analysis

Cultured cells and mouse tissues were lysed employing radio immunoprecipitation assay (RIPA) lysis buffer (Beyotime Biotech, Shanghai, China) supplemented with a protease and phosphatase inhibitor cocktail (Sigma-Aldrich), following established procedures [60]. Nuclear protein extracts were generated using the NE-PER Nuclear and Cytoplasmic Extraction Kit from Thermo Fisher Scientific, following the manufacturer’s instructions.

Equal amounts of protein were separated using sodium dodecyl sulfate–polyacrylamide gel electrophoresis (SDS-PAGE) and subsequently transferred onto a polyvinylidene difluoride (PVDF) membrane. These membranes were blocked with TBST containing 5% (w/v) skim milk for 1 h at room temperature. Subsequently, they were incubated overnight at 4 °C with the following primary antibodies: Lats1 (1:1,000; Cell Signaling Technology), phospho-Smad1/5 (1:1,000; Cell Signaling Technology), GAPDH (1:10,000; Bioworld), Smad1/5 (1:1,000; Cell Signaling Technology), Smad4 (1:1,000; Cell Signaling Technology), phospho-Stat3 (1:1,000; Cell Signaling Technology), Stat3 (1:1,000; Cell Signaling Technology), YAP (1:1,000; Santa Cruz Biotechnology), phospho-YAP (1:1,000; Cell Signaling Technology), TAZ (1:1,000; Cell Signaling Technology), MOB1 (1:1,000; Cell Signaling Technology), phospho-MOB1 (1:1,000; Cell Signaling Technology), and Lamin A/C (1:1,000; Cell Signaling Technology). The next day, the membranes were incubated in the appropriate secondary antibodies, and the signals were visualized using Pierce ECL Western Blotting Substrate.

### Hematological parameters and iron measurements

Hematological parameters were assessed at the Animal Experiment Center of Zhejiang University employing a hematology analyzer (Sysmex). Serum iron, transferrin saturation, and tissue non-heme iron were determined following previously established protocols [60].

### ChIP assay

ChIP was carried out utilizing the Simple ChIP Plus Enzymatic Chromatin IP Kit (#9005; Cell Signaling Technology), following the manufacturer’s guidelines. Immunoprecipitation was conducted with magnetic beads and antibodies specific to immunoglobulin G (IgG; Cell Signaling Technology), acetyl-histone H3 (Lys9) (Cell Signaling Technology), or YAP (Cell Signaling Technology). The retrieved DNA fragments were utilized directly for quantitative RT-PCR analysis, employing primers specifically designed for amplifying the *Hamp1* promoter (refer to Table S2).

Where indicated, Huh7 cells were treated with XMU-MP-1 or DMSO for 12 h, and then collected and analyzed using ChIP as described above using antibodies against IgG (Cell Signaling Technology) or YAP (Cell Signaling Technology). The recovered DNA fragments were used directly for quantitative RT-PCR using primers designed to amplify the TEAD-binding sites in the human *HAMP* or mouse *Hamp* promoter (Table S3).

### Co-IP assays

To immunoprecipitate endogenous YAP, STAT3, SMAD4, and TAZ, Huh7 cells were treated with XMU-MP-1 or DMSO for 12 h. The cells were then lysed using RIPA buffer (Beyotime Biotech) containing protease and phosphatase inhibitor cocktail (Sigma-Aldrich) as described previously [60]. For immunoprecipitation, the cell lysates were incubated with monoclonal antibodies against YAP or SMAD (Cell Signaling Technology) overnight at 4 °C. On the following day, 40 μl of protein A/G agarose beads (Santa Cruz Biotechnology) were added, and the samples were gently rotated at 4 °C for 4 h. The beads were harvested by centrifugation at 3,000 rpm for 5 min and subsequently subjected to 3 washes with phosphate buffer solution (PBS) buffer. Following the final wash, the proteins were denatured by boiling in 2× loading buffer. The proteins were then detected using Western blot analysis with YAP, STAT3, SMAD4, and TAZ antibodies (Cell Signaling Technology) as described above.

### Immunofluorescence

Huh7 cells were treated for 12 h with RGFP966 (Selleck Chemicals, Houston, TX) or DMSO, fixed in 4% paraformaldehyde, and then stained using an anti-YAP antibody (1:100, Cell Signaling Technology) as the primary antibody and Cy3-labeled Goat Anti-Rabbit IgG (H+L) (1:1,000, Beyotime) as the secondary antibodies.

### Statistical analysis

Unless specifically stated otherwise, all summary data are expressed as the mean ± standard error of the mean. Group differences were evaluated using either a one-way or two-way analysis of variance with Tukey’s multiple comparison test or the Student’s *t* test, as appropriate. Statistical significance was defined at *P* < 0.05.

## Data Availability

Data are available upon request to F.W. (fwang@zju.edu.cn).
