# Supplementary figures and images for: Hepatic HDAC3 Regulates Systemic Iron Homeostasis and Ferroptosis via the Hippo Signaling Pathway
Source: Research (Wash D C). 2023 Nov 30;6:0281. doi: 10.34133/research.0281 (PMC10687581; doi:10.34133/research.0281)

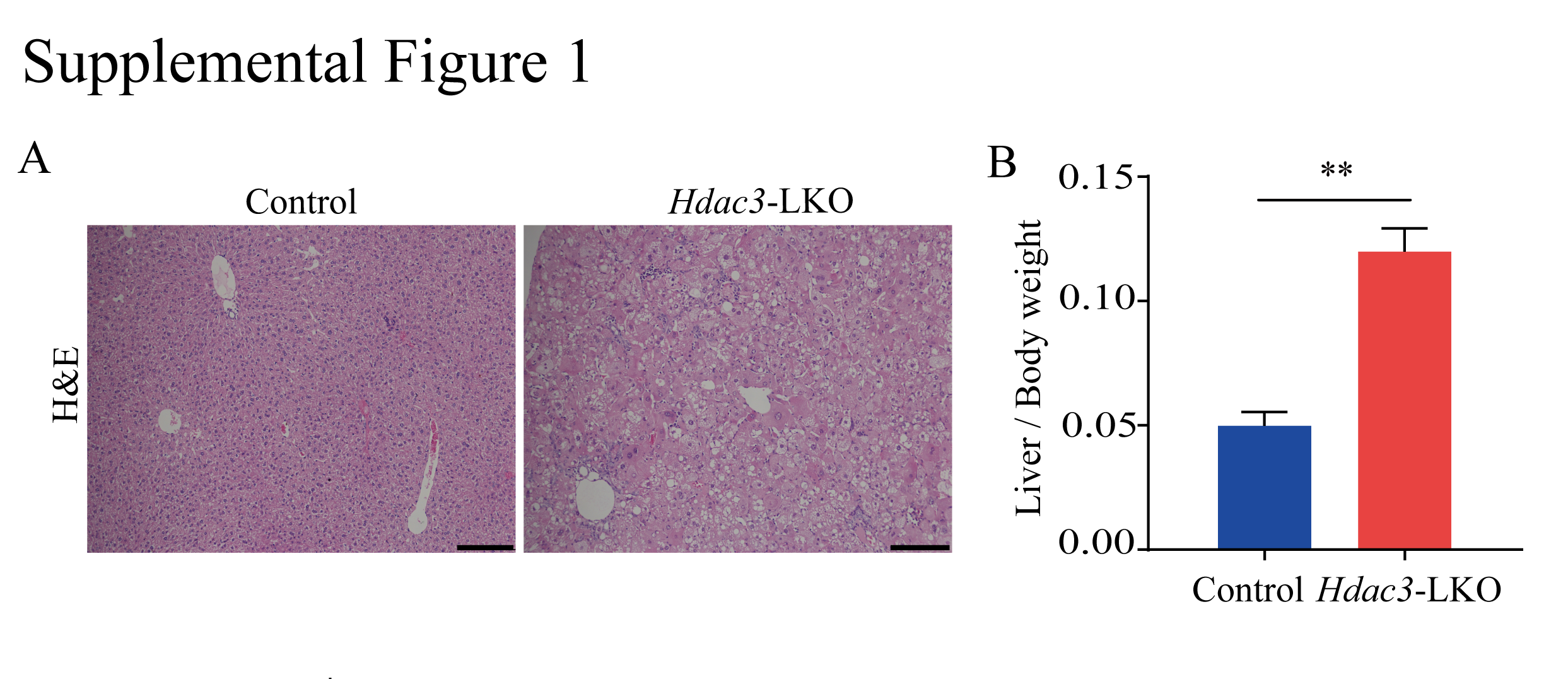

Supplement: Supplementary 1 — Figs. S1 to S6 Tables S1 to S3 [file research.0281.f1.zip › Supplemental Figure 1.tif]

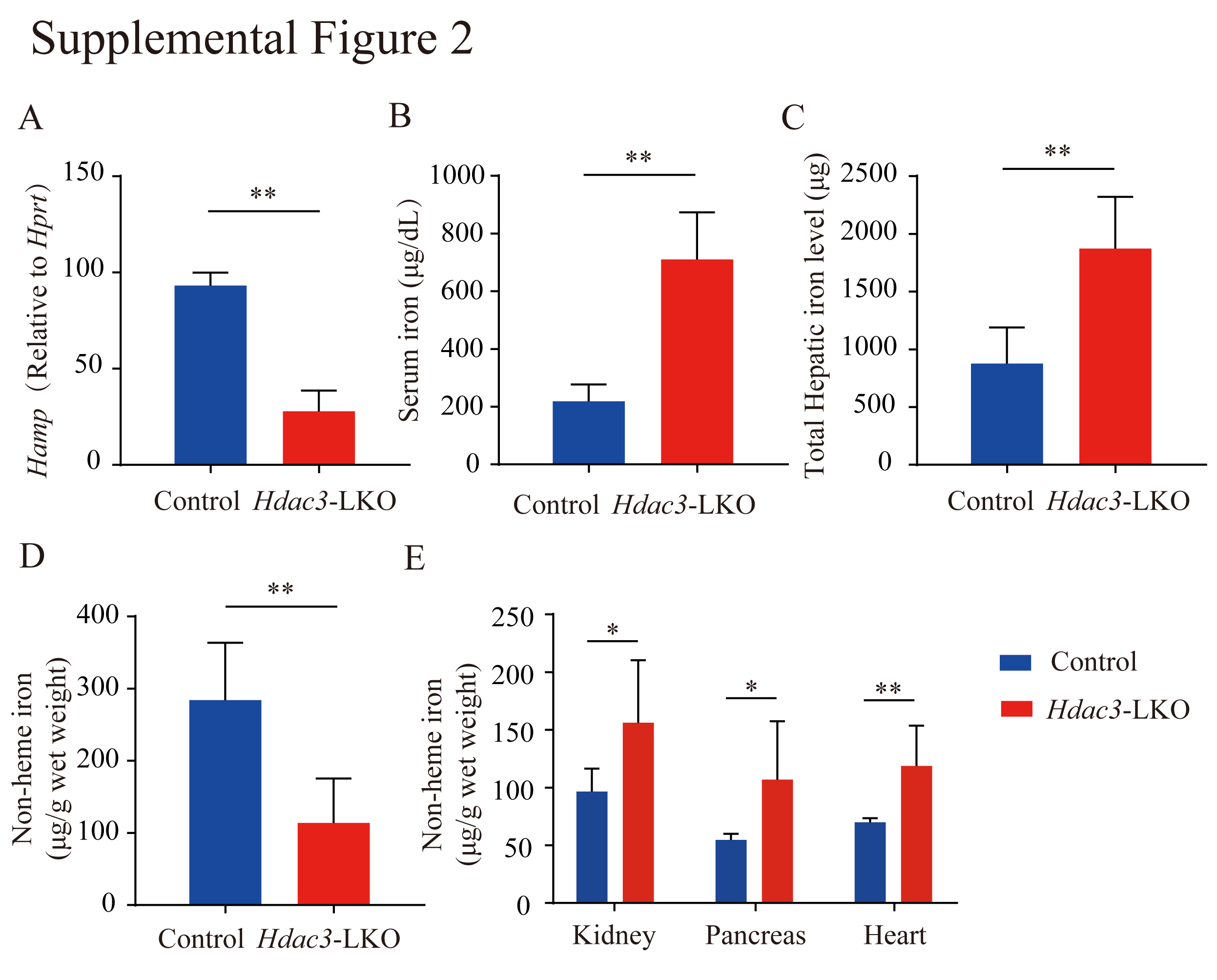

Supplement: Supplementary 1 — Figs. S1 to S6 Tables S1 to S3 [file research.0281.f1.zip › Supplemental Figure 2.tif]

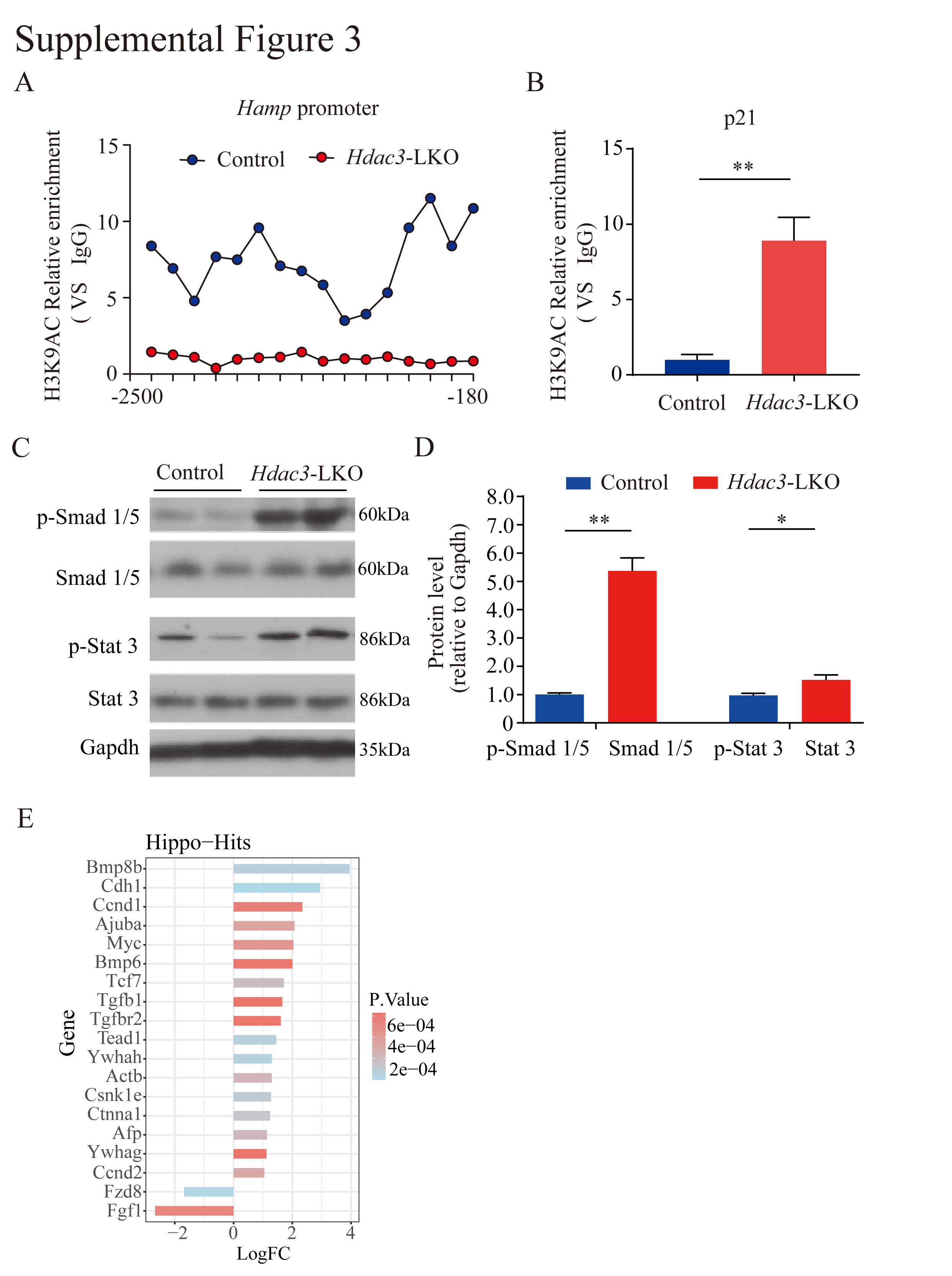

Supplement: Supplementary 1 — Figs. S1 to S6 Tables S1 to S3 [file research.0281.f1.zip › Supplemental Figure 3.tif]

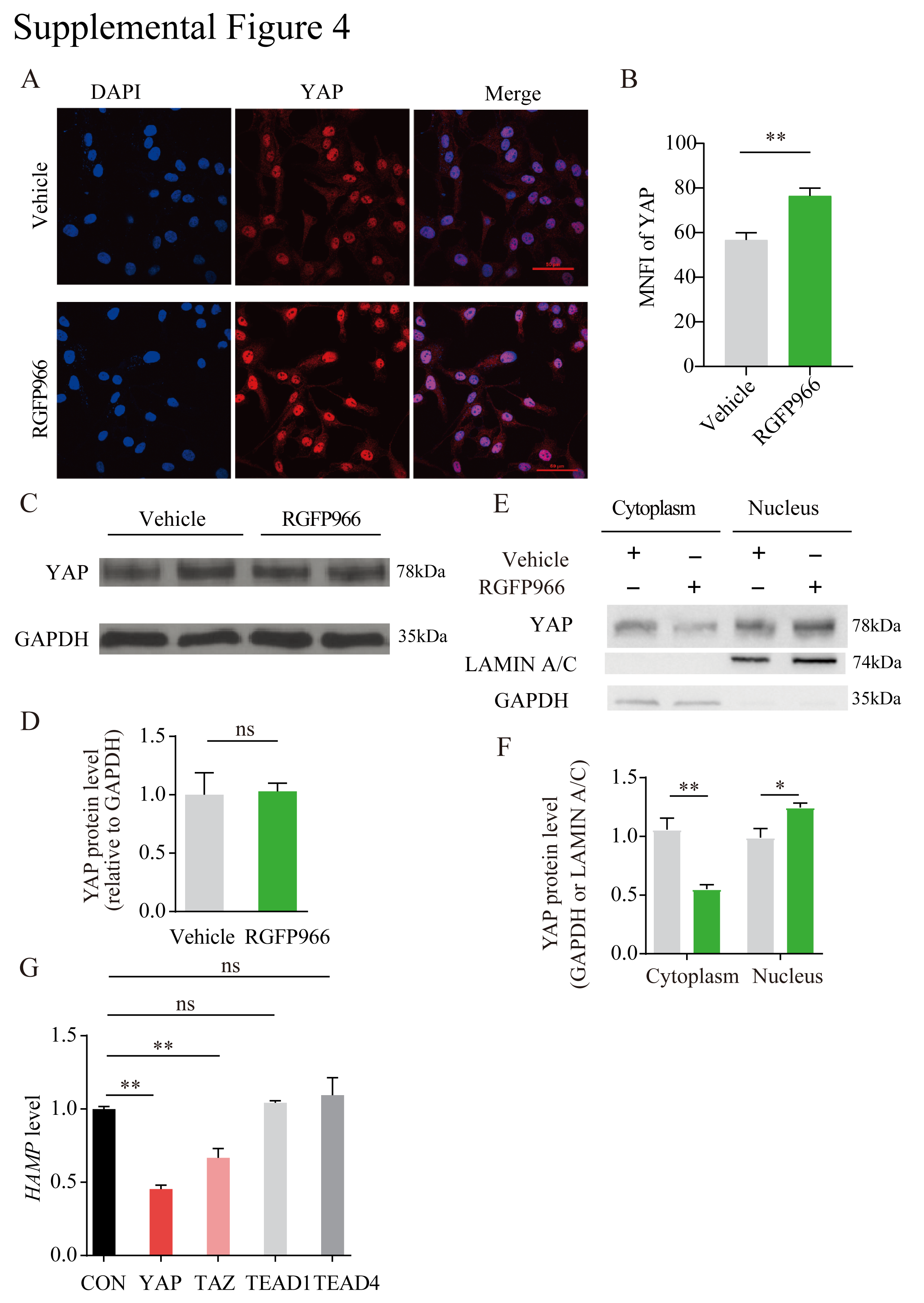

Supplement: Supplementary 1 — Figs. S1 to S6 Tables S1 to S3 [file research.0281.f1.zip › Supplemental Figure 4.tif]

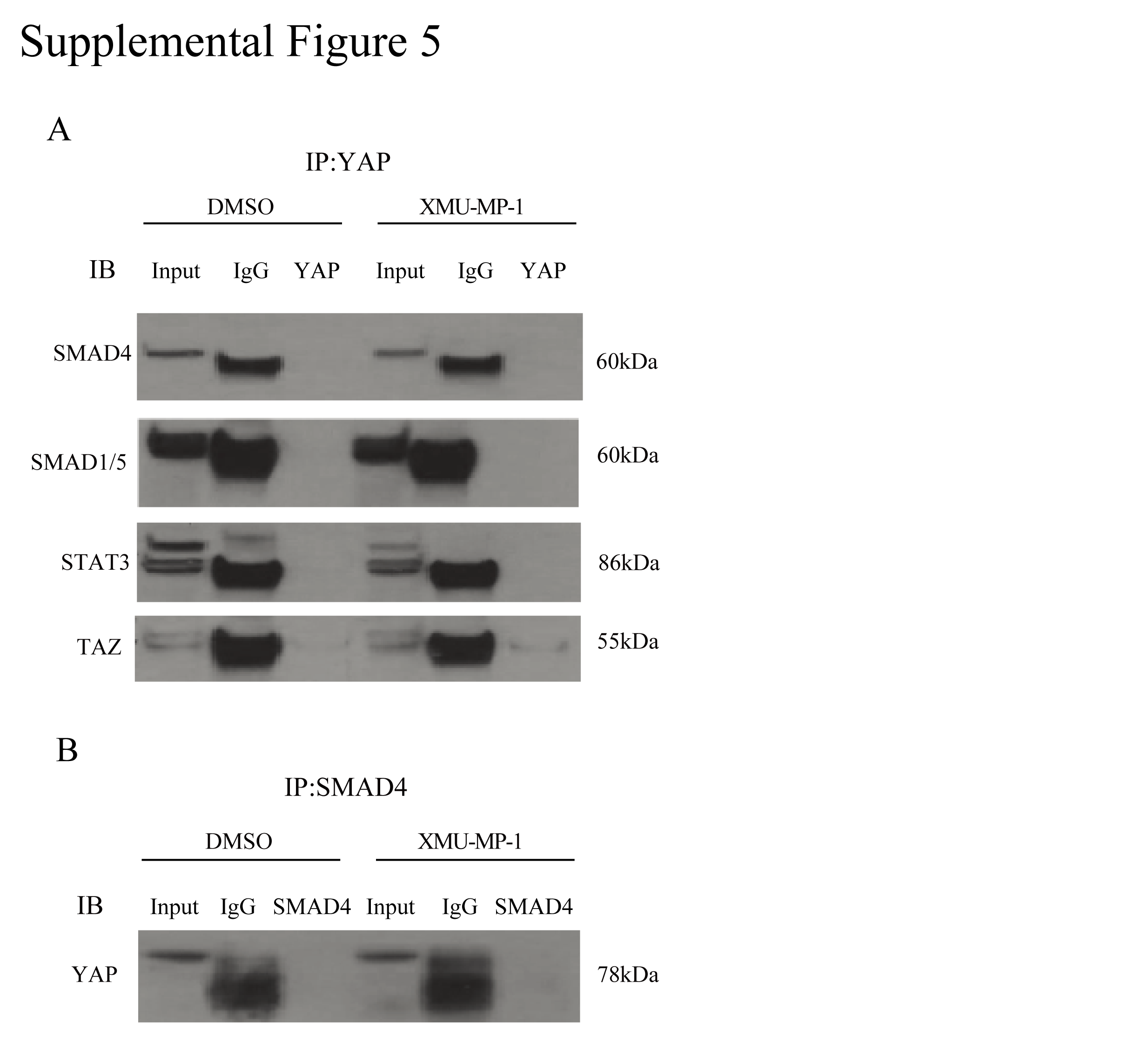

Supplement: Supplementary 1 — Figs. S1 to S6 Tables S1 to S3 [file research.0281.f1.zip › Supplemental Figure 5.tif]

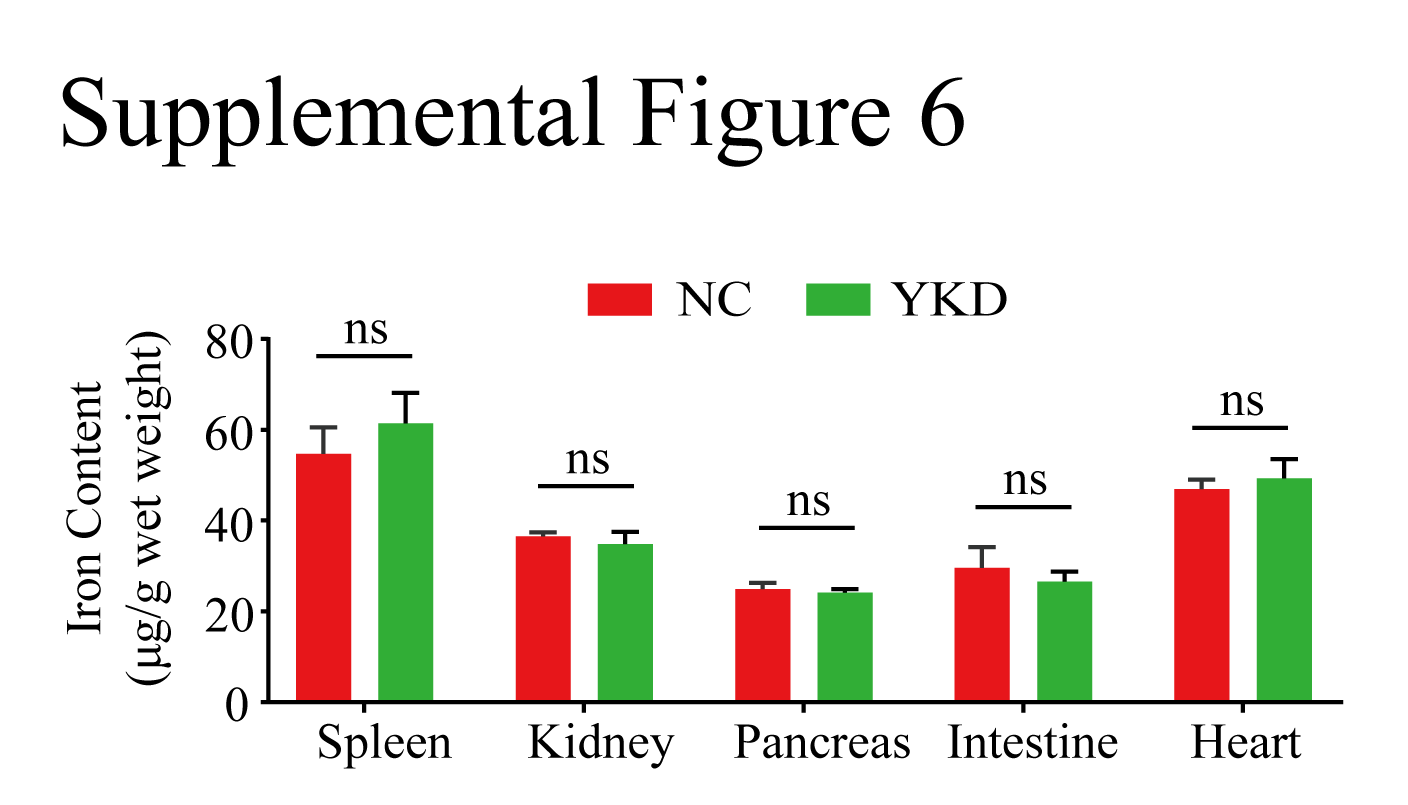

Supplement: Supplementary 1 — Figs. S1 to S6 Tables S1 to S3 [file research.0281.f1.zip › Supplemental Figure 6.tif]
