# Supplementary material for: Hepatic HDAC3 Regulates Systemic Iron Homeostasis and Ferroptosis via the Hippo Signaling Pathway
Source: Research (Wash D C). 2023 Nov 30;6:0281. doi: 10.34133/research.0281 (PMC10687581; doi:10.34133/research.0281)
Supplement: Supplementary 1 — Figs. S1 to S6 Tables S1 to S3 [file research.0281.f1.zip › RESEARCH_R1_Meng et al SM.doc]

**Supplementary Materials for**

**Hepatic HDAC3 regulates systemic iron homeostasis and ferroptosis via the Hippo signaling pathway**

Hongen Meng^1#^, Yingying Yu^1#^, Enjun Xie^1#^, Qian Wu^1^, Xiangju Yin^2^, Bin Zhao^3^, Junxia Min^1*^, Fudi Wang^1*^

^1^The Second Affiliated Hospital, The First Affiliated Hospital, Institute of Translational Medicine, School of Public Health, Zhejiang University School of Medicine, Hangzhou, China

^2^Institute of Emergency Management, Henan Polytechnic University, Jiaozuo, China

^3^MOE Key Laboratory of Biosystems Homeostasis & Protection, Zhejiang Provincial Key Laboratory for Cancer Molecular Cell Biology, and Innovation Center for Cell Signaling Network, Life Sciences Institute, Zhejiang University, Hangzhou 310058, China

^#^ Hongen Meng, Yingying Yu, and Enjun Xie contributed equally to this work.

**Correspondence**

Fudi Wang, The Second Affiliated Hospital, School of Public Health, Zhejiang University School of Medicine, Hangzhou, China. Email: [fwang@zju.edu.cn](mailto:fwang@zju.edu.cn)

Junxia Min, The First Affiliated Hospital, Institute of Translational Medicine, Zhejiang University School of Medicine, Hangzhou, China. Email: [junxiamin@zju.edu.cn](mailto:junxiamin@zju.edu.cn)

**This file includes:**

**Supplemental Figures S1 to S6**

**Supplemental Tables S1 to S3**

**Supplemental Figure S1.** **Liver histology and liver weight in *Hdac3*-LKO mice and control mice.** (A) Liver sections were prepared from 8-week-old *Hdac3*-LKO mice and control littermates and stained with H&E. Scale bar = 200 μm**.** (B) Summary of the liver/body weight ratio of 8-week-old *Hdac3*-LKO and control littermates; n≥6 mice per group. ***p*<0.01 (Student’s *t*-test).

**Supplemental Figure S2.** **Summary of hepatic *Hamp* mRNA and non-heme iron levels** **in Hdac3-LKO mice and control mice.** Hepatic *Hamp* mRNA (A), serum iron (B), hepatic iron (C), splenic non-heme iron (D), and non-heme iron in the kidney, pancreas, and heart (E) measured in *Hdac3*-LKO and control littermates that were fed a high-iron diet; n≥6 mice per group. **p*<0.05 and ***p*<0.01 (Student’s *t*-test).

**Supplemental Figure S3. Evaluation of potential regulatory mechanisms involving Hdac3-regulated *Hamp* expression.** (A-B) H3K9AC ChIP followed by quantitative RT-PCR of the *Hamp* promotor (A) and *p21* promoter (B) in 8-week-old *Hdac3*-LKO mice and control littermates, expressed relative to control IgG. (C-D) Western blot analysis (C) and quantification (D) of the indicated proteins in the canonical pathways that regulate Hamp. (E) Summary of differentially expressed genes in the Hippo pathway in *Hdac3*-LKO mice versus control littermates. **p*<0.05 and ***p*<0.01 (Student’s *t*-test).

**Supplemental Figure S4. HDAC3 inhibitor RGFP966 upregulates YAP signaling pathway in Huh7 cells.** (A) Fluorescence images of Huh7 cells treated with 80 μM RGFP966 or vehicle and immunostained for YAP; the nuclei were counterstained with DAPI. (B) The mean nuclear fluorescence intensity (MNFI) values were plotted for YAP. Each confocal microscopy image is a representative of three independent biological replicates. The bar graphs represent MNFI values ± SEM from three independent biological replicates. Scale bar = 50 μm. (C and D) Western blot analysis (C) and quantification (D) of YAP measured in untreated and RGFP966-treated Huh7 cells. (E and F) Western blot analysis (E) and quantification (F) of YAP measured in the cytoplasm and nucleus of untreated and RGFP966-treated Huh7 cells. (G) Relative *HAMP* mRNA levels measured in Huh7 cells expressing the indicated proteins, normalized to control (CON). **p*<0.05, ***p*<0.01, and ns, not significant (one-way ANOVA with Tukey’s post hoc test).

**Supplemental Figure S5. Co-IP of YAP and SMAD4 in Huh7 cells with or without treatment of XMU-MP-1.** (A) Huh7 cells were treated with XMU-MP-1 (10 μM) or vehicle (DMSO) for 12 h; the cells were then lysed, and a YAP antibody was used for IP, followed by western blot analysis of the indicated proteins. (B) Similar to (A), except a SMAD4 antibody was used for IP, followed by western blot analysis of YAP.

**Supplemental Figure S6. Iron content in YKD-treated mice.** At 5 weeks of age, *Hdac3*-LKO received an i.v. injection of pAAV-TBG-shYap1 or pAAV-TBG-NC (10^11^ vg/g); 4 weeks later, the mice were sacrificed, and iron content was measured in the indicated tissues. ns, not significant (Student’s *t*-test).

**Supplementary Table S1. Sequences of the primers used for quantitative PCR analysis**

| Gene | Forward primer (5’ 🡪 3’) | Reverse primer (5’ 🡪 3’) |
| --- | --- | --- |
| *HPRT1* | TTCCTTGGTCAGGCAGTATAATC | TGGCTTATATCCAACACTTCGT |
| *HAMP* | CAGCTGGATGCCCATGTTC | CAGCAGCCGCAGCAGAA |
| *YAP* | CAGGTTGGGAGATGGCAAAG | GGGCTGTGACGTTCATCTGG |
| *CYR61* | TGCCGCCTTGTGAAAGAAAC | CTCAAACATCCAGCGTAAGTAAACC |
| *TAZ* | GTGCTGGAAAAAGAAGAGAGAAAGG | GCAGGATGATGGGGTTGAGA |
| *CTGF* | TTCCAAGACCTGTGGGATGG | GGGAGTACGGATGCACTTTTTG |
| *Hprt* | TTTCCCTGGTTAAGCAGTA | TGGCCTGTATCCAACACTTCGAGA |
| *Hamp* | GCACCACCTATCTCCATCAAC | TTCTTCCCCGTGCAAAGG |
| *Tmprss6* | TTGCTGGTCTTGGCTGCGCT | AATGACGGTTGAGCACCCGGAG |
| *Smad7* | CTCCTGCTGTGCAAAGTGTT | GATTCACAGCAACACAGCCT |
| *Hfe* | TGTGAGGTGCATGAAGACAACAG | TCTTGCCCGTCATAACCATATCT |
| *Hjv* | CCAGGCTGAGGTGGACAATC | GTCGGTCGCCCCCATT |
| *Il-6* | TGTATGAACAACGATGATGCACTT | ACTCTGGCTTTGTCTTTCTTGTTATCT |
| *Taz* | TCCTATGACGTGACCGACGA | GGGTCTTGCCATGTGGTGAT |
| *Yap* | CAGTGTCTTCTCCTGGGATGTCTC | GGTGCTTTGGCTGATGGTGTC |
| *Ctgf* | GGACACCTAAAATCGCCAAGC | ACTTAGCCCTGTATGTCTTCACA |

**Supplementary Table S2. Sequences of the primers used for ChIP analysis of the mouse *Hamp* promoter**

| Location | Forward primer (5’ 🡪 3’) | Reverse primer (5’ 🡪 3’) |
| --- | --- | --- |
| -2590 - -2410 bp | CCGGCTCACCCTTCAGC | GGCATCGTTTTCGCATGGAG |
| -2400 - -2244 bp | ATCTGTCCATCAGCCTCCCT | AAGCTCCCTCCTTCCCATGA |
| -2255 - -2038 bp | AGGAGGGAGCTTTGGGGAT | TTGCAGTTAAATACCTCTCTCTCT |
| -2061 - -1901 bp | AGAGAGAGAGGTATTTAACTGCAA | TTAATGCCCCCAGCCATTGT |
| -1918 - -1767 bp | AATGGCTGGGGGCATTAACT | GCCCTCATCATTGGCTGGTA |
| -1777 - -1615 bp | ATGATGAGGGCTGGCCTTG | CTGTGGCTTCTCAGGACAGG |
| -1580 - -1461 bp | CCCCAAGAGATTGGCTCACC | GGGTTCTCAAGACACCAGGG |
| -1476 - -1237 bp | GGTGTCTTGAGAACCCTGTCTCG | TTTGGAAGTGGTAAGGCCAAAGA |
| -1264 - -1054 bp | CCCTGTCTTTGGCCTTACCA | CCTTGGTATGAGCAGCCTGA |
| -1069 - -934 bp | GCTGCTCATACCAAGGGGTC | CCCATGCCACTTGGACAACA |
| -953 - -802 bp | TGTTGTCCAAGTGGCATGGG | GGCTTGACGATGTATTCCTCAAAT |
| -727 - -537 bp | ACTTAGAGGCCAAGTAGCACCA | GCTGTCATGTTGGATTCCCCTTCT |
| -541 - -349 bp | ACAGCTACATCATATGGTCTTCACA | GTGGAAGAACTGTACCTTTACCC |
| -334 - -204 bp | ACTGTTTAGGGGAAAGAAGGGG | GTCACCTACAGCCAGCAGAG |
| -182 - -48 bp | GTTCCCGCTTATCTCTCCCG | TCTAGGAGCCAGTCCCAGTC |
| -137 - -8 bp | TCAGAGCAAAATGGGGGTGG | TCTGTTCTGCTGTGCAGTCT |

**Supplementary Table S3. Sequences of the primers used for YAP-ChIP-quantitative RT-PCR of the human *HAMP1* and mouse *Hamp1* promoter TEAD-binding sites**

| Gene | Forward primer (5’ 🡪 3’) | Reverse primer (5’ 🡪 3’) |
| --- | --- | --- |
| *hTEAD Binding site 1* | ACTTGGCTGACACTGGATGG | GCCTGACTGCAGTGCCTT |
| *hTEAD Binding site 2* | AAGTGGTTCAGCAACCAGGA | CACCACTAGCTGGGGGATGA |
| *hTEAD Binding site 3* | AGTGGGGAGGGGGCCT | TAGGGCCTCAAAGGGGACAT |
| *mTEAD Binding site 1* | GGGGTCGGGGAATACCATC | TCGAGACAGGGTTCTCAAGAC |
| *mTEAD Binding site 2* | CTGACTGGATGCTCCCTGTG | CCTTGGTATGAGCAGCCTGA |
